# Facilitators and barriers to health enhancing physical activity in individuals with severe functional limitations after stroke: A qualitative study

**DOI:** 10.3389/fpsyg.2022.982302

**Published:** 2022-10-19

**Authors:** Leah Reicherzer, Markus Wirz, Frank Wieber, Eveline S. Graf

**Affiliations:** ^1^Institute of Physiotherapy, School of Health Sciences, ZHAW Zurich University of Applied Sciences, Winterthur, Switzerland; ^2^Institute of Public Health, School of Health Sciences, ZHAW Zurich University of Applied Sciences, Winterthur, Switzerland; ^3^Social Psychology and Motivation Lab, Department of Psychology, University of Konstanz, Konstanz, Germany

**Keywords:** health enhancing physical activity/activities, stroke, behavior change wheel (BCW), capability, opportunity, motivation–behavior (COM-B), theoretical domains framework (TDF)

## Abstract

**Background:**

Patients with chronic conditions are less physically active than the general population despite knowledge of positive effects on physical and mental health. There is a variety of reasons preventing people with disabilities from achieving levels of physical activities resulting in health benefits. However, less is known about potential facilitators and barriers for physical activity (PA) in people with severe movement impairments. The aim of this study was to identify obstacles and facilitators of PA in individuals with severe disabilities.

**Materials and methods:**

Using a qualitative approach to explore individuals’ subjective perspectives in depth, five community-dwelling adults (age 52–72, 2 female, 3 male) living with chronic mobility impairments after stroke that restrict independent PA were interviewed. A semi structured topic guide based on the theoretical domains framework was utilized. The interview data was analyzed thematically, and the theoretical domains framework constructs were mapped onto the main and sub-categories.

**Results:**

The six main categories of facilitators and barriers along the capability, opportunity, motivation–behavior (COM-B) framework were: (1) physical capabilities, (2) psychological capabilities, (3) motivation reflective, (4) motivation automatic, (5) opportunity physical, and (6) opportunity social. The physical capabilities to independently perform PA were variable between participants but were not necessarily perceived as a barrier. Participants were highly motivated to maintain and/or increase their abilities to master their everyday lives as independently as possible. It became clear that a lack of physical opportunities, such as having access to adequate training facilities can present a barrier. Social opportunities in the form of social support, social norms, or comparisons with others can act as both facilitators and barriers.

**Conclusion:**

While confirming known barriers and facilitators that impact the ability of individuals with functional limitations to be active, the findings highlight the need and opportunities for comprehensive service models based on interdisciplinary collaborations.

## Introduction

Physical activity (PA) is defined as any bodily movement produced by skeletal muscles that requires energy expenditure (WHO, 2010). Being physically active is associated with health benefits and contributes to the cure and prevention of a variety of non-communicable diseases (NCD), such as heart disease and stroke as well as cancer and diabetes. PA also contributes to the prevention of NCD risk factors such as hypertension, overweight, and obesity and is associated with improved mental health, a delay in the onset of dementia, improved quality of life, and wellbeing (WHO, 2020). Given the powerful effects of PA, the term Health Enhancing Physical Activity (HEPA) has been established.

Patients with chronic conditions are less physically active than the general population (de Hollander and Proper, 2018). With only about 21% of individuals attaining the recommended PA, stroke is associated with the lowest prevalence of recommended PA (Brawner et al., 2016; Aguiar et al., 2017; Kang et al., 2021). For non-ambulatory patients with stroke, it is even more difficult to adhere to recommended PA levels (Lloyd et al., 2018).

Habitual PA can positively influence secondary comorbidities that often accompany severe chronic conditions (Durstine et al., 2000). Guidelines recommend similar doses of PA for persons with chronic conditions as for the general population and emphasize the importance of PA for the secondary prevention, e.g., of recurrent cardio-vascular events (Billinger et al., 2014; Pfeifer and Geidl, 2017). As patients with neurological conditions are at a higher risk of experiencing adverse events such as falls or cardiac events during exercise, pre-exercise assessment and tailored exercise programs are needed, also to promote long-term adherence (Billinger et al., 2014).

Approaches such as the Physical Activity for People with Disability (PAD) model relate PA to functioning and disability and outline determinants of PA behavior in people with disability (van der Ploeg et al., 2004). They highlight environmental and personal factors that independently as well as interacting with each other influence the individual’s behavior. Shields et al. (2012) divided barriers into four categories: Personal, Social, Environmental, Policy and Programs. For stroke, studies have shown that motivation, anxiety, beliefs about capabilities, environmental context and resources, and social influences modify engagement in PA (Damush et al., 2007; Nicholson et al., 2014; Thilarajah et al., 2020). The most commonly reported barriers were environmental (access, transport, and cost), health problems and stroke related impairments while the main motivator was social support (connecting with other stroke survivors) (Nicholson et al., 2013). A conceptual framework on PA after stroke illustrates the relationship between motivation (desire to be active) and capability (resources to be active), and the related influencing factors, such as the direct effects of stroke as a barrier or social support through health professionals or other survivors as a facilitator (Morris et al., 2017).

A comprehensive framework to understand, predict, and change individuals behavior is the behavior change wheel (BCW) (Michie et al., 2011). It differentiates three levels that contribute to behavior: a behavior system, intervention functions, and policy categories. It includes personal, social, environmental, as well as policy and program factors that have been outlined as main sources of barriers and facilitators in the PA models (van der Ploeg et al., 2004; Shields et al., 2012) but also addresses how to derive tailored interventions. The BCW structures the development of behavior change interventions in three different stages: understanding the behavior, identifying intervention options, and identifying content and implementation options. Within the first stage, it employs the so-called capability, opportunity, motivation–Behavior (COM-B) model as core of the behavior system to understand the behavior in context. This acronym stands for individuals’ Capabilities (physical and psychological), Opportunities (physical and social), and Motivation (reflective and automatic) as determinants of Behavior. It can be complemented by the theoretical domains framework (TDF) (Cane et al., 2012). The TDF consists of 14 domains that represent determinants of adherence to a behavior or behavior change, mapped onto the overarching COM-B model. Despite existing best-practice examples (Connell et al., 2016; Hall et al., 2020a,b) and the recommendations to develop interventions underpinned by behavior change theory as they are more effective than non-theoretical interventions (Craig et al., 2008) and allow testing the specific determinants of behavior and refining the intervention (Davis et al., 2015), the aforementioned tools and processes have not been applied to understand and change PA in individuals with severe disabilities.

Therefore, the current research aims to explore facilitators and barriers to PA in individuals with severe functional limitations using the BCW framework to inform how an intervention should look like that optimally supports individuals with severe disabilities to attain the HEPA recommended PA.

## Materials and methods

### Study design

A descriptive design with a qualitative approach, underpinned by the COM-B framework was chosen. Semi-structured interviews were used for data collection. All names have been replaced by pseudonyms.

### Sampling and recruitment

German-speaking adults with chronic neurological mobility limitation were purposively recruited through staff from outpatient rehabilitation facilities in Switzerland. Inclusion criteria were: severe, chronic (>6 months) mobility limitation that restrict independent PA, no or mild cognitive impairment, and no additional diseases that prevent from PA. The sampling aim was to represent individuals from different age groups, gender, and severity of the condition where possible. If participants expressed interest, the research team informed them about the study and appointments at locations convenient to the participant were scheduled. Table 1 provides an overview of study participants and their characteristics.

**TABLE 1:** Participant characteristics.

	Age	Gender	Occupational status	Living situation	Time since stroke	Walking abilities
B1	59	M	Part-time work (coaching rehabilitation patients and relatives)	Living alone	19 years	Ambulatory with walking aid on level surface (FAC 3–4) for short distances
B2	59	F	Invalidity pension	Living with husband	2 years	Ambulatory with walking aid on level surface (FAC 3–4) for short distances
B3	55	F	Invalidity pension	Living with her children (14–20 years old)	8 years	Ambulatory with walking- aid or supervision for stairs (FAC 4–5)
B4	72	F	Retired	Living with husband (also has a disability)	12 years	Ambulatory with walking aid on level surface (FAC 3–4) for short distances
B5	72	M	Retired	Living alone	N/A	Ambulatory including stairs (FAC 5)

FAC, functional ambulation category; M, Male; F, Female.

### Data collection

A semi structured topic guide was developed based on the TDF (Cane et al., 2012). Each of the 14 domains has been addressed in the topic guide (Supplementary file) by one to five main questions and prompts, aimed at eliciting participants beliefs and experiences surrounding capability, opportunity, and motivation in detail. The topic guide was pilot tested in a previous study and adapted to the present study population. Interviews were held by two experienced members of the research team, a physiotherapist (female, LR) and a psychologist (male, FW). No prior relationship between participants and researchers had been established. The interviews were carried out at the rehabilitation clinics, in a café close to the participants home or by telephone between March and May 2022. They were audio recorded and lasted between 20 and 100 min.

### Data analysis

All interview recordings were summarized and relevant parts (i.e., opinions on PA, descriptions of PA behavior, and mentioning of influences on PA behavior) were transcribed verbatim. Thematic analysis involves familiarization with the text trough repeated reading, identification of codes and synthesis in thematic categories (Kuckartz, 2014). Coding was guided by the interview guideline (COM-B), but inductive identification of codes and categories was applied when unanticipated topics were found in the material. The authors (LR and FW) discussed the codes and categories to achieve consensus over the results. Finally, the TDF constructs were mapped onto the definite main categories and subcategories, and another consensus discussion followed. Quotes are used in the following section to illustrate the categories.

## Results

**FIGURE 1:**
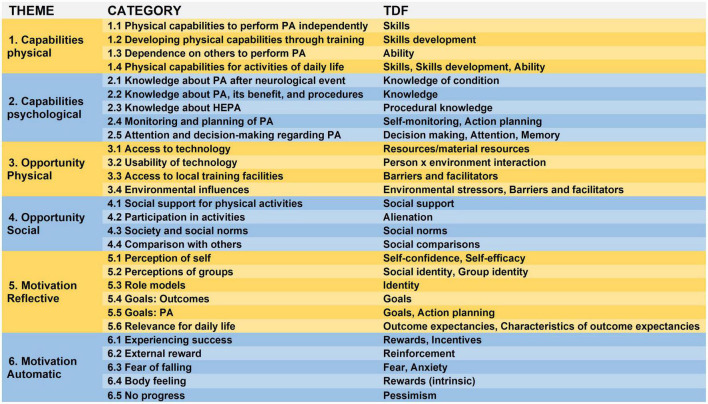
The six themes and 28 categories from the thematic analysis of the interviews and the theoretical domain framework (TDF) constructs. HEPA, health enhancing physical activity; PA, physical activity; TDF, theoretical domain framework.

Interview findings were summarized into 6 main- and 23 subcategories. Figure 1 presents the TDF constructs mapped onto the main and subcategories.

### Physical capabilities

Participants described different **physical capabilities to independently perform PA** (skills). Important for participants was the ability to walk, *“I can walk like a normal person” (B5).* Limited or no upper extremity function on the affected side limited activities such as swimming. Paresis of the foot flexor was reported as a limitation for walking independently. Over time, participants **developed physical capabilities through training** (skills development):

*“I can remember, when I tried to go to the supermarket for the first time, 200 m from my house, I took the rollator and it took 2 h. But I have kept working on it, kept trying, and later I could walk with a walking stick” (B4)*.

Participants report different levels of **dependence on others to perform PA** *(abilities):* One participant relied on assistance for transportation to the training facility and for the transfer to training devices. PA itself could be performed individually according to all participants. A central theme for participants were their **physical capabilities for daily life activities** (skills and abilities). They all used daily life activities to describe and quantify their physical capabilities. For example, one participant when asked about his muscle strength, answered: “*Well it’s enough to do my daily routines on my own” (B1).*

### Psychological capabilities

Some participants had acquired **knowledge about PA after stroke** (knowledge of condition) and gave examples of consequences of inactivity in wheelchair users, i.e., the loss of core strength. They were aware that a training program tailored to the individual capabilities is warranted. In **general, knowledge about PA, its benefits and procedures** (knowledge) pertained to its importance, the negative consequences of inactivity and the positive effects on for example the vascular system:

*“It’s really important because everything moves when you move. I’m thinking circulation of the blood. And if you do not move, there is muscle atrophy” (B4).*

The term HEPA was not familiar to participants neither were official PA recommendations (**knowledge about HEPA**). Most participants instead had ideas about how much PA would be good or relevant for themselves, ranging from short sessions every day to three times per week 1.5 h. Three participants engaged in some form of **PA monitoring and planning** (self-monitoring and action planning), for example through apps. One participant described planning the amount and frequency of her PA:

*“I do make plans. For example that every day I walk two rounds in the morning and then cycle for half an hour in the evening” (B2).*

Accounts of participants demonstrated **attention and decision-making regarding PA**. One participant described using exercises for the arm as a strategy to relieve shoulder pain.

### Opportunity physical

Several external factors affect participants opportunities to engage in PA. One of them was the **access to technology** (Resources/material resources). It emerged that having access to the latest technology beyond the acute phase of treatment was difficult. One participant described that her rehabilitation clinic has technologies she would like to use, but availability is an issue as the demand is high. Another participant was interested in a robotic device to practice arm movements, but it was too costly to purchase for home use. This theme is linked to a second environmental factor: **access to local training facilities** (barriers and facilitators). According to the interviews, there are not enough facilities that are easy to reach for outpatients and offer the right equipment for wheelchair users. For participants who train with technologies, **usability** (person x environment interaction) can be an issue. One participant who trains regularly with a robotic device described discomfort:

*“(*…*) you are secured with a harness. And it is not very comfortable, so somewhere it always pinches or cuts in” (B2).*

Another participant who uses an app for training reported having difficulties navigating through the app. **Environmental influences** (environmental stressors, barriers, and facilitators) were a strong determinant for participants in their decision or ability to be physically active. Bad weather presented an obstacle for many because it reduces the motivation to go out, can present a physical barrier (i.e., icy conditions for wheelchair users), but can also impact physical functioning:

*“Cold temperatures affect my bladder* (…*) so it’s really depending on the weather for me, sunshine gets me further” (B1).*

Public infrastructure, such as escalators or stairs, affected participants differently. They offer training opportunities for balance and walking ability but also make moving around difficult. Small details, like having handrails on both sides, can make a difference:

*“(*…*) you always have to go up and down the stairs and that’s always been a challenge for me, step up, step down, and then nothing to hold on to–that was really hardcore” (B3).*

### Opportunity social

**Social support** can make PA possible as the stories of participants demonstrate: *“I also have very good friends who help me. For example, we went canoeing once and I didn’t even know if I could get in and then they said, no problem, we’ll help you” (B3).*

Social support included direct help from friends or families to execute a certain activity like walking, canoeing or tandem cycling, but also assistance to get to training locations. Some participants reported training together with a friend with a similar condition. PA served to stay connected with others and **participate in society** (alienation):

*“Colleagues went up the mountain, into the snow, and then I have to say, okay, I just wasn’t there, that stresses me out.” (Inquiry by interviewer: is it more about being a part of it, or about missing out on the activity?) “Actually, it’s about the social aspect, I realize that before I was always there and now, I’m a bit on the outside” (B3).*

**Social norms were** another enabler or barrier. Generally, participants believed social norms or society do not influence their PA behavior. However, they spoke about insecurities of how they were perceived in public, when for example being out with their partners.

*“When we’re somewhere… (me) sitting in a wheelchair. I always say, do they think the poor woman or do they think the poor man” (B2)*.

Regarding **comparison with others** (social comparisons) participants compared themselves with someone who is less active as a negative example but also with someone with a similar condition who has different abilities. The following statements illustrates the latter, but also shows how this person puts it in perspective and is aware, that not all comparisons are suitable or beneficial:

*“I know a woman who suffered a stroke at the same time, and she is very diligent. She’s very diligent, she goes swimming. But I can’t swim because of this arm. (…). I am jealous, of this woman. I would also like to go swimming and I tried, but it was difficult. And she now, she walks at home. But, I can’t compare, everyone is different” (B4).*

### Motivation reflective

One theme of reflective motivation was the **perception of self** (self-confidence and self-efficacy): having overcome obstacles helped to build confidence in one-self to be active. One participant described: *“I just needed these obstacles to know, I can do this” (B3).* Participants also stated that a group that trains and is active together could be good for motivation and would be beneficial for people with mobility impairment, i.e., their **perceptions of groups** (group-identity) could be a motivating factor:

*“A lot of people with handicap distance themselves from others, because they have a handicap and these people have to be brought together” (B1)*.

In most interviews, participants brought up a **role model** (identity), somebody that has influenced them in their decision to be active: *“if this man can do it, then maybe I can also” (B5)*. Participants described two different types of goals: **goals pertaining to outcomes** (goals) and **goals for PA** (goals and action planning). Outcome oriented goals included end or intermediate results participants wanted to achieve, either relating to body functions, such as regaining muscle function of the foot flexor, or mobility improvements, such as being able to climb stairs.

*“It was my goal to at least come home on weekends and then I knew, I had to be able to climb the stairs and I have to perform transfers, that was indeed a motivation” (B2)*.

Goals for PA were process-oriented, such as walking around the house or training a set amount of time daily. A theme that emerged in all interviews was the **relevance for daily life** (outcome expectancies): The wish to perform daily life activities independently was a strong motivator for many participants and PA or training was described as intertwined with daily life.

*“If I feel like, this is good for me, I have a training for my everyday life, then that really motivates me” (B1).*

### Motivation automatic

**Experiencing success** (rewards and incentives) motivated participants to keep up their training and to be active. Examples were reaching a goal or a smaller milestone and being able to see progress over time: *“If I’ve had a small experience of success, then I am more motivated”(B3).* One participant described how a stalled or **no progress** toward her goals has impeded her motivation to train regularly (pessimism).

“*It’s simply my decision (whether to train or not), but I feel like in the beginning the curve has gone up massively and then later the curve flattens and is even a little bit worse, so I think this is part of my decision somehow” (B3).*

**External rewards** (reinforcements) were mentioned as a motivating influence, such as the affirmation of a care giver or feedback given by a device after a workout. For participants, the **fear of falling** (fear and anxiety) had an influence on their ability to be active, especially when being at home alone or outside. One participant recounted how she had fallen while being home alone and has had great difficulties to stand up without someone’s help. *“Falling would actually be a nightmare for me” (B3)*. Training or physical therapy elicited an improved **body feeling** (intrinsic rewards) in some participants, which they describe as “it makes you feel good.” Others did not observe any changes in how they feel after or during exercise, or described that they feel very tired.

## Discussion

This study explored facilitators and barriers to PA in individuals with severe functional limitations, using the comprehensive BCW framework with the COM-B model and TDF. Five semi-structured interviews revealed barriers and facilitators along the six main categories of the COM-B, namely individuals’ physical and psychological capability, their physical and social opportunities, as well as their reflective and automatic motivation. The TDF constructs were mapped onto the results of the thematic analysis, providing us with the key behavioral constructs to consider for an intervention.

Participants were highly motivated to be independent in everyday life and have developed and applied strategies to pursue that goal. PA was important to achieve that goal. Aspects of autonomous motivation also played a role. Although detailed knowledge on HEPA was limited, we discovered a general understanding of the importance of PA and knowledge about stroke relating to psychological capabilities and that a lack of physical opportunities, such as having access to adequate training facilities can present a barrier. Finally, social opportunities in the form of support, social norms, or comparisons with others were found to positively influence PA behavior.

**FIGURE 2:**
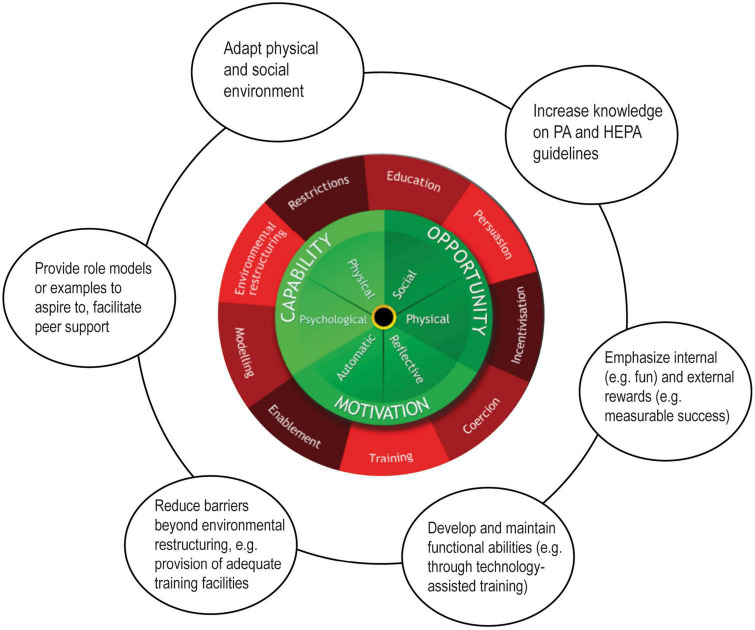
The behavior change wheel BCW and possible intervention strategies in the context of this study. Figure based on “The behavior change wheel” by Michie et al. (2011) licensed under CC BY 2.0 (https://creativecommons.org/licenses/by/2.0/).

These findings complement and confirm other research on individuals with disabilities such as the PAD model by van der Ploeg et al. (2004) or Shields et al. (2012). However, the BCW approach, representing a synthesis of several models, expands other approaches by allowing to identify interventions to tackle the respective barriers and outlining policy categories that can be leveraged to bring these interventions about (see Figure 2). Complementary, the TDF constructs mapped onto the results of the thematic analysis present a link between behavior change theories and techniques of behavior change. The Behavior Change Technique Taxonomy (BCTT, V1) (Michie et al., 2013) is a classification system that allows characterizing the active content of interventions and understand the mechanisms behind behavioral changes such as specific if-then plans (Wieber et al., 2015). We argue that the BCW provides a well-suited framework to address the complexity of the challenges that individuals with functional limitations face to attain HEPA and to develop and implement tailored interventions.

The need to provide persons with mobility restrictions access to HEPA is evident. They are exposed to an increased risk of NCD and relapses of neurological events due to the forced sedentary lifestyle. NCD affect both, physical and mental wellbeing and thus have an impact on health-related quality of life. One element in modifying these risks is regular PA. If those affected want to reduce the health risks through self-determined PA, there are hardly any adapted training options available to them. However, health enhancing PA in people with severe mobility impairment has not been sufficiently studied yet. These persons are therefore at a disadvantage in terms of health, as they cannot take advantage of preventive PA. As age is the single most important risk factor for stroke (Sacco et al., 1997), the demographic development point to an increasing number of such cases in the future, thereby increasing the need to develop effective solutions to provide HEPA to everyone.

Whereas rehabilitation technologies such as a robot for the walking training have proven to enable intensive and effective functional training in persons with impaired mobility (Mehrholz et al., 2017; Wirz et al., 2017) they can also be used to achieve the recommended amount of HEPA and positively affect the determinants of behavior outlined in the BCW and the TDF. For example, technology can provide reinforcement through external rewards in the form of positive feedback for good performance. The results of the present study contribute to the investigation of the potential of rehabilitation technologies to assist people with mobility limitations to become physically active on a regular basis in order to minimize the risk of inactivity-associated conditions.

New PA-oriented services for individuals with mobility restrictions are needed as well as their evaluation. To date, there are a few studies who have addressed this topic. However, sample sizes were small and the long-term effect of PA training has not been studied yet. Also, it remains unclear for how long the effects of training persist after discontinuation of the training. The literature supports the assumption that a training with rehabilitation devices, which enable large movements of multiple body segments, results in immediate physiological responses (Lefeber et al., 2017). Moreover, detailed knowledge about the reasons for (non-) participation in PA and the amount of PA performed inform interventions and strategies for health promoting PA.

A successful and promising training program also holds the potential to be scaled up to other populations. For example, people with progressive diseases or children with neurologic impairments could benefit from comprehensive health service models. Both are populations with unique challenges and requirements, needed to be addressed specifically.

### Methodological considerations

The small sample size, the mapping of TDF-constructs by one researcher and the susceptibility of interviews to social desirability limit the informative value of the results. Although more interviews might have allowed to reach saturation, patients from different rehabilitation clinics and centers ensure geographical variety. Moreover, techniques like peer debriefing or triangulation would have further strengthened the validity of the COM-B and TDF framework based analyses but as consistency has been reviewed with a second researcher, the findings still inform the first stage of the BCW approach for intervention development.

Individuals with mobility impairments are a very heterogeneous group. Even though the diagnosis is the same, the symptom presentation after rehabilitation vary greatly (Dunn et al., 2016). In this study, the severity ranged from moderate, corresponding to a functional ambulation category (Holden et al., 1984) of 4–5 to severe, corresponding to a functional ambulation category of 2–3. This challenges adequate tailoring of intervention components and identifying appropriate outcome measures to evaluate the effectiveness of an intervention. Often, the time lag between intervention and expected health outcomes is considerable, and thus difficult to capture in a trial. However, given the increased health risks of individuals with mobility impairments, these difficulties do not justify delaying our efforts to develop effective evidence-based programs that allow individuals with mobility impairments to get access to the HEPA benefits.

## Conclusion

The COM-B and TDF framework allowed a comprehensive analysis of the facilitators and barriers for HEPA in individuals with functional limitations. Although they might not know the specific HEPA suggestions, they are aware of the importance of PA and they are strongly motivated to maintain and/or increase their physical abilities to master their everyday lives as independently as possible and to be socially engaged. Despite these facilitators, barriers such as the lack of access to training facilities–without burdening one’s social network–keep them from HEPA and call for changes in the training models that are offered. Thus, health service models for individuals with functional limitations to sustainably attain the HEPA suggestions are needed as they represent promising opportunities to foster behavior change and reduce social inequalities. Central stakeholders are called upon to join their forces to develop and implement these models.

## Data availability statement

The raw data supporting the conclusions of this article will be made available by the authors, without undue reservation.

## Ethics statement

Ethical review and approval was not required for the study on human participants in accordance with the local legislation and institutional requirements. Written informed consent for participation was not required for this study in accordance with the national legislation and the institutional requirements.

## Author contributions

EG, MW, and FW developed the research question and designed the interview guideline. FW and LR collected the data. LR analyzed the data. All authors contributed to the writing the manuscript and read and approved the final manuscript.
