# Supplementary material for: Facilitators and barriers to health enhancing physical activity in individuals with severe functional limitations after stroke: A qualitative study
Source: Front Psychol. 2022 Oct 19;13:982302. doi: 10.3389/fpsyg.2022.982302 (PMC9628747; doi:10.3389/fpsyg.2022.982302)
Supplement: Supplementary file 1 [file Table_1.DOCX]

**Interview Questions Health Enhancing Physical Activity**

| **TDF domains** | **Interview questions** |
| --- | --- |
| Opportunity physical (COM-B) | |
| Skills | What physical capabilities do you currently have that enable you to be physically active?   - What do these abilities look like in terms of strength? Mobility? - Are you able to walk? What level of support do you need? - (For which activities) Do you need physical support from care support (CS)? What does this support look like? - How can the CS contribute to the maintenance and development of physical abilities? |
| Opportunity psychological (COM-B) | |
| Knowledge | What do you know about the theory of PA/training?   - How is your training structured? - What intensity should be used for training? (possibly how can this be recorded?). How often should training take place? - What must be considered when using the training equipment? What steps are necessary for operation? |
|  | Do you know what HEPA is?   - Current HEPA recommendations? Do you know other recommendations? - Do you think PA recommendations are important and why? - Do you think regular PA has benefit? |
| Memory, Attention and Decision Processes | Do or did you notice changes in your activity patterns over time? |
|  | For what reasons do you decide to start exercising?  Or: What keeps you from engaging in PA? (Did you consciously decide against PA?)   - Can you name reasons for which you started or stopped with PA? Possibly also a specific event? - What encourages or influences that decision? - How do others, for example family, influence the decision? |
| Behavioral regulation | What are reasons for you to be more or less physically active (feeling good, pain, not feeling well etc.)?   - How do difficult situation, such as being stressed, ill, distracted or sad, feeling weak, fearful, affect your PA? Can you still be active? - Do you often feel in the mood to exercise? |
|  | What helps or hinders your planning?  Are there unavoidable situations that keep you from training? What are they? e.g. pain |
| Motivation Reflective (COM-B) | |
| Social/Professional Role & Identity | Has PA changed the structure of your daily life?   - Which aspects have changed? (Changed schedule...)   Has your self-confidence changed as a result of you exercising? |
|  | Do you know others with similar impairments, that are physically active? Or have you heard from others?  Do you feel differently perceived by others when you are active (compared to when you are not)? |
|  | Does it motivate you when others are active too?   - How would a group influence your motivation to be physically active? Would you exercise more? - Do groups like this exist close to you? |
| Beliefs about Capabilities | What influences how self-confident you feel?   - Do you feel confident when you’re physically active? |
|  | Have you ever been discouraged from exercising by others?   - Did this make you feel insecure?   How do you deal with unexpected disruptions during PA?  How can you motivate yourself to train?  Do you think you can have an impact on your health with your behaviour? Is it positive? |
|  | How is your self-confidence strengthened by/during PA?  Can you motivate others to be active? |
| Optimism | How do you feel during the training?  Do you feel comfortable/confident?  How do you feel afterwards? |
|  | Has your self-confidence changed as a result of you exercising?  Do you feel differently perceived by others when you are active (compared to when you are not)? |
| Beliefs about Consequences | What effects do you think regular PA has? On the body? Mentally? |
| Goals | What goals do you have regarding your PA?   - How often do you want to exercise? For how long? - What level of activity do you want to achieve? - Longer term: goals regarding ability to walk? |
| Intentions | What do you think changes as a result of training?  What changes do you expect in terms of strength/mobility, ability to walk? |
| Motivation Automatic (COM-B) | |
| Reinforcement | What do you think are rewards for the effort of exercising, if any? |
| Emotion | Is there anything you are afraid of in training?  (e.g. not enough strength but also longer term things like arthritis).   - How can/could you overcome this fear? - Are others afraid for you when you train? |
|  | How do you feel during the training?   - Do you feel comfortable/confident? - How do you feel afterwards? |
| Opportunity Physical (COM-B) | |
| Environmental context and resources | Do weather or terrain have an influence on your motivation or ability to train at all? What influence? Why? |
|  | Do you have access to training facilities, equipment etc.?   - If you needed new equipment (or to replace old equipment), would it be possible to get it? - Could you afford it? - What help would you need to get it? |
|  | What are the environmental barriers to carrying out training?  What environmental factors help in the execution of training? |
| Opportunity Social (COM-B) | |
| Social influences | Do others put pressure on you to be more active? If yes, why do you think they do that?  Do you also feel social pressure to be active? If yes, how do you react to that? |
|  | Would you like more (social) support? What kind? On a societal level?  Who is supporting you to be physically active? How?  Are you dependent on others in order to do your PA?  What reaction do you expect from friends/family etc. if you don't want to train anymore or want to start training? |
|  | Do you feel there are societal norms around the topic? How do they affect you?  To what extent do you think other stroke survivors are physically active? |
